# How policymakers value end-of-life treatments for rare and common diseases in China: evidence from a contingent valuation study

**DOI:** 10.1186/s41256-025-00434-w

**Published:** 2025-08-26

**Authors:** Han Cheng, Shan Jiang, Taoran Liu, Boyang Li, Shanquan Chen, Ao Li, Hao Chen, Haiyin Wang, Yuanyuan Gu

**Affiliations:** 1 Macquarie University Centre for the Health Economy, Macquarie Business School and Australian Institute of Health Innovation, Macquarie University, Sydney, NSW Australia; 2 Department of Infectious Diseases and Public Health, City University of Hong Kong, Hong Kong, China; 3 School of Political Science and Public Administration, Wuhan University, Wuhan, Hubei China; 4 International Centre for Evidence in Disability, London School of Hygiene and Tropical Medicine, London, UK; 5 West China School of Public Health and West China Fourth Hospital, Sichuan University, Chengdu, China; 6 School of Medicine and Health Management, Tongji Medical College, Huazhong University of Science and Technology, Wuhan, China; 7 Shanghai Health Development Research Center (SHDRC), Shanghai, China

**Keywords:** Willingness to pay, Priority setting, Healthcare resource allocation, Contingent valuation, Drug reimbursement, Rare disease, QALY, Cost-effectiveness threshold

## Abstract

**Background:**

Understanding policymakers’ value judgements in reimbursement decisions is essential for promoting equity and guiding informed healthcare decision-making. This study aimed to estimate and compare Chinese policymakers’ willingness-to-pay (WTP) per quality-adjusted life year (QALY) specifically in end-of-life treatment scenarios involving life-threatening common and rare diseases.

**Methods:**

We conducted a contingent valuation study employing single-bounded dichotomous-choice questions among 120 experts formally appointed by China’s National Healthcare Security Administration to serve on the National Reimbursement Drug List Expert Committee in recent years. Participants evaluated hypothetical scenarios describing end-of-life treatments providing a one-QALY gain for patients with life-threatening common or rare diseases. Data were collected primarily through face-to-face interviews, supplemented by online responses when in-person meetings were impractical. Statistical analysis was performed using probit regression models, and t-tests were conducted to compare WTP values between scenarios.

**Results:**

A total of 99 policymakers participated. Participants’ WTP per QALY for end-of-life treatments in common disease scenarios ranged from CNY 78,031 (0.98 times GDP per capita) to CNY 126,449 (1.58 times GDP per capita). In contrast, WTP was significantly higher for rare diseases, ranging from CNY 183,392 (2.29 times GDP per capita) to CNY 219,691 (2.75 times GDP per capita). Analysis of individual characteristics revealed that female participants and those with expertise in pharmacoeconomics exhibited significantly higher WTP values in common disease scenarios (*p* < 0.05), though these factors had varied effects in rare disease scenarios.

**Conclusions:**

This study provides novel estimates of Chinese policymakers’ WTP per QALY specifically in end-of-life contexts involving common and rare diseases, highlighting the significant impact of disease rarity on reimbursement decisions. These findings offer empirical support for adopting differentiated cost-effectiveness thresholds tailored to end-of-life treatments based on disease rarity in China.

**Supplementary Information:**

The online version contains supplementary material available at 10.1186/s41256-025-00434-w.

## Introduction

Treating rare diseases presents significant challenges for healthcare priority setting, particularly in end-of-life care [1]. These challenges arise from the intersection of high treatment costs, limited clinical evidence, and heightened ethical complexity. Therapies for rare diseases, especially those targeting life-threatening conditions in end-of-life scenarios, are often extremely expensive due to the small populations they serve and the high fixed costs of research and development [1]. This raises concerns about the sustainability of allocating substantial healthcare resources to treatments that benefit relatively few individuals, especially when such allocations may displace more cost-effective interventions [2]. In addition, many rare diseases lack robust clinical trial data, and evidence on long-term outcomes is often sparse or uncertain. This uncertainty complicates the evaluation of treatment effectiveness and limits the applicability of standard cost-effectiveness criteria [3]. In end-of-life contexts, where treatments may offer only modest extensions of life or quality of life improvements, policymakers must also consider societal preferences that often support compassionate care, even when such interventions do not meet conventional cost-effectiveness benchmarks. These issues underscore a fundamental tension in health policy between the goals of equity and efficiency. On one hand, there is a moral imperative to ensure access to potentially life-saving treatments regardless of rarity or cost. On the other hand, limited healthcare budgets require trade-offs that prioritize interventions generating the greatest overall health benefits. As a result, priority setting for rare diseases at the end of life often involves difficult and contested judgments about value, fairness, and the appropriate role of public funding.

Cost-effectiveness analysis is a central tool for guiding treatment coverage and pricing decisions [4]. A cost-effectiveness threshold represents the maximum amount a health system is willing to pay for a unit of health gain, typically measured as a quality-adjusted life year [5]. Thresholds are intended to reflect either society’s monetary valuation of health improvements, known as the demand-side perspective, or the health opportunity costs of displacing existing services, known as the supply-side perspective [6]. Earlier thresholds were often based on arbitrary benchmarks, such as the widely cited fifty thousand United States dollars per quality-adjusted life year or the World Health Organization’s recommendation of one to three times gross domestic product per capita [7, 8]. More recent approaches advocate empirically grounded estimates tailored to local contexts [9].

While cost-effectiveness thresholds have traditionally been applied uniformly, there is increasing recognition of the need for flexibility to reflect public values and ethical considerations, particularly in cases involving end-of-life care and rare conditions [1, 10]. In the United Kingdom, the National Institute for Health and Care Excellence allows for higher thresholds in these contexts [11]. For example, some drugs for rare diseases have been accepted for funding with incremental cost-effectiveness ratios as high as one hundred thousand to three hundred thousand pounds per quality-adjusted life year, well above the standard thresholds applied to more common diseases [12]. These policy adjustments suggest that society may be willing to pay more for treatments in circumstances perceived to be especially urgent or morally compelling.

Despite these global developments, limited empirical evidence exists on how Chinese policymakers value health gains in end-of-life scenarios, particularly when comparing rare and common conditions. Some studies have estimated thresholds for common diseases in China using stated preference approaches, such as the contingent valuation method, discrete choice experiments, and analyses based on the value of statistical life [5, 6, 13]. However, these studies do not include rare diseases or explicitly consider end-of-life contexts, limiting their relevance for current policy debates. This study addresses a critical evidence gap by eliciting the willingness-to-pay (WTP) of Chinese policymakers for quality-adjusted life year gains in life-threatening end-of-life scenarios. We compare their valuations for treatments targeting rare and common diseases using the contingent valuation method. By focusing explicitly on decision-makers and on health technologies in end-of-life care, this study provides timely and policy-relevant insights to inform more equitable and sustainable reimbursement decisions in China.

## Methods

### Participants

The target population consisted of 120 experts appointed by China’s National Healthcare Security Administration (NHSA) to the National Reimbursement Drug List Expert Committee over recent years. These experts constitute the entire population of national-level policymakers involved in drug reimbursement decisions. All were selected for their expertise in health economics, health insurance, pharmacy, or clinical medicine.

We conducted a contingent valuation study to elicit policymakers’ WTP for a QALY gain in scenarios involving rare and common diseases. Data collection occurred between May and July 2023, primarily via in-person interviews (approximately 85%), supplemented by video conferencing where geographic constraints existed. Interviews followed a standardized protocol, and interviewers received training to ensure participants fully comprehended the hypothetical scenarios, remained engaged throughout the valuation process, and provided thoughtful responses. Interviewers also monitored non-verbal indicators, such as confusion or fatigue, to enhance response quality. A potential limitation is that the study focuses only on national-level policymakers; thus, perspectives from provincial policymakers or broader professional backgrounds may differ. However, our approach aligns directly with the study objective of capturing perspectives from those making national reimbursement decisions, thereby ensuring relevance to policy contexts.

### Contingent valuation question

The survey consisted of two parts. The first part collected background information on participants, including sex, work experience (< 5 years, 5–10 years, 11–20 years, or > 20 years), area of expertise (health insurance, pharmacy, pharmacoeconomics, or other), and geographic affiliation (province and region). These variables were incorporated as covariates in the Probit models to examine their influence on WTP for a QALY in a common or rare disease context, enabling the identification of characteristics associated with variations in WTP. Provinces were grouped regionally into northern, southern, eastern, and western China for regional analyses.

**Fig. 1:**
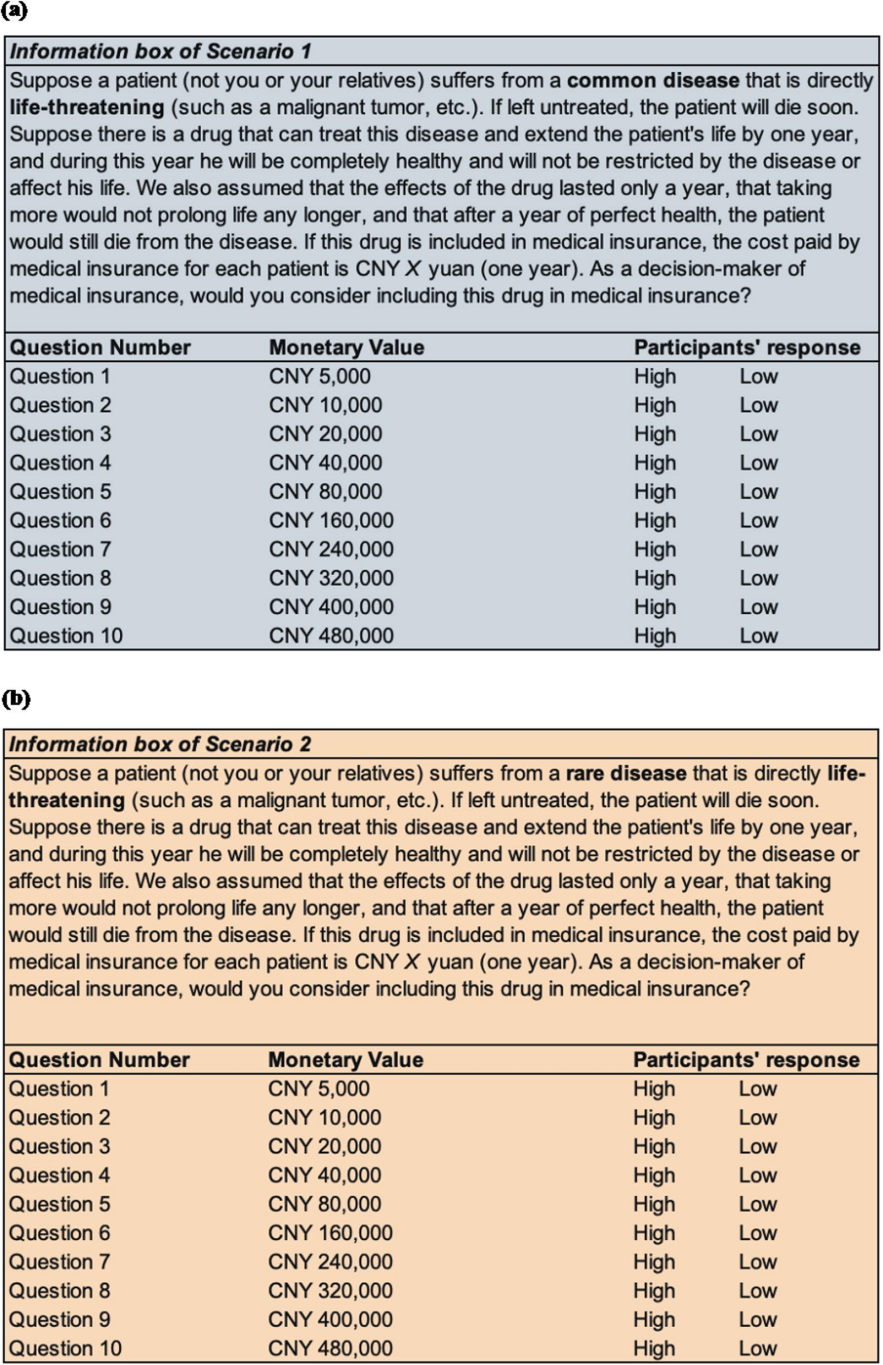
Information and questions presented to participants. Panel (**a**) presents the information box of scenario 1, in which participants were required to assume including a drug in medical insurance for treating a common life-threatening disease. Panel (**b**) represents the information of scenario 2, in which participants were required to assume including a drug in medical insurance for rare life-threatening diseases.

In the second survey part, contingent valuation questions consisted of twenty single-bounded, closed-ended dichotomous choice questions. Following the methodology by Shiroiwa et al. [14] we developed two hypothetical disease scenarios, each containing ten bid questions (Fig. 1). Scenario 1 described a common life-threatening disease, emphasizing that a proposed drug would extend life by one year of perfect health, after which the patient would inevitably succumb to the disease. Participants indicated whether this drug should be included in national public health insurance at specified annual coverage costs ranging from 0.0625 to six times China’s 2022 GDP per capita (CNY 80,976). Participants responded sequentially to ascending bid amounts until indicating a bid was “High,” ending the questioning sequence.

Scenario 2 followed an identical procedure; however, in this scenario, the hypothetical patient was described as suffering from a rare disease. The scenarios were deliberately constructed to be otherwise identical, ensuring that the only difference between Scenario 1 and Scenario 2 was the disease’s rarity. This design allowed us to isolate and measure participants’ responses specifically to disease rarity, controlling for all other factors.

Participants were explicitly instructed to represent a societal perspective, rather than an individual perspective, to align the contingent valuation method with societal WTP evaluations.

### Quality control

To minimize response errors or misunderstandings inherent in contingent valuation studies, several quality control measures were implemented. Interviewers were trained to detect potential response errors through participants’ non-verbal cues, such as hesitation or confusion. Upon detection, interviewers neutrally reconfirmed participant responses to ensure accurate representation of true preferences.

To maintain participant concentration, interviews occurred in quiet, distraction-free settings, with online interviewees instructed to minimize environmental distractions. Periodic checks for fatigue and clarifying questions helped sustain participant engagement. Standardized explanations ensured consistent presentation and comprehension of contingent valuation scenarios and bid questions across all interviews.

Following data collection, we evaluated the responses to ensure logical consistency. Given that the format presented bid values in ascending order, a rational response pattern would involve indicating WTP (“High”) up to a participant’s maximum threshold, and “Low” for all higher bid values—thus reflecting a monotonic preference structure. We systematically screened for inconsistencies, such as instances where a participant indicated WTP for a higher bid after previously rejecting a lower bid, or vice versa. Such patterns violate the principle of monotonicity, which assumes that participants are less likely to pay higher amounts for the same benefit.

Responses exhibiting these inconsistencies were flagged for further review. We then assessed whether such deviations could be attributed to identifiable factors or if they reflected genuine data quality concerns. If logical inconsistencies could not be explained or rectified, the corresponding responses were deemed invalid and excluded from subsequent analyses. This validation procedure was essential to ensure that the dataset accurately captured participants’ true WTP, thereby providing a robust foundation for estimating cost-effectiveness thresholds.

### Statistical analysis

Descriptive statistics summarized participant characteristics, and frequencies were calculated to present WTP intervals (e.g., [$WTP_{j}$, $WTP_{j + 1}$], where $j \in \left[ {1, 9} \right]$ is the question number in each scenario).

Probit models were estimated, coding “Low” responses as 1 and “High” as 0. The probit model was preferred over logit due to its alignment with the assumptions of random utility theory and its frequent use in health-related contingent valuation literature [14]. Participants’ WTP was modelled as:

1 $$\begin{array}{*{20}c} {WTP_{i} = \beta x_{i} + \varepsilon_{i} , \varepsilon_{i} \sim N\left( {0, \sigma^{2} } \right)} \\ \end{array}$$

where the WTP of the participant $i$ is composed of two main parts: $\beta x_{i}$ represents the deterministic part (consisting of the vector of parameters ($\beta$) and the vector of explanatory variables) and $\varepsilon_{i}$ the stochastic part of WTP. When participants were presented with the particular bid amount, they would answer “yes” if their WTPs were greater than the bid amount; otherwise, they would answer “no”. Therefore, the probability of observing a WTP that was greater than the bid amount is formulated as:

2 $$\begin{array}{*{20}c} {\Pr \left( {response_{i} = 1{|}x_{i} } \right) = \Pr \left( {WTP_{i} > Bid_{i} } \right) = \Pr \left( {\beta x_{i} + \varepsilon_{i} > Bid_{i} } \right) = \Pr \left( {\varepsilon_{i} > Bid_{i} - \beta x_{i} } \right)} \\ \end{array}$$

Therefore, we have the probability of positive WTP as:

3 $$\begin{gathered} \Pr \left( {response_{i} = 1{|}x_{i} } \right) = \Pr \left( {v_{i} > \frac{{Bid_{i} - \beta x_{i}{\prime} }}{\sigma }} \right) \hfill \\ \Pr \left( {response_{i} = 1{|}x_{i} } \right) = 1 - \phi \left( {\frac{{Bid_{i} - \beta x_{i}{\prime} }}{\sigma }} \right) = \phi \left( {x_{i}{\prime} \frac{\beta }{\sigma } - Bid_{i} \frac{1}{\sigma }} \right) \hfill \\ \end{gathered}$$

where the $\phi \left( x \right)$ represents the standard cumulative normal.

Using the Probit model, we estimated the coefficients for explanatory variables (e.g., sex, working experience, affiliation locations, expertise) in the model $\alpha = \frac{\beta }{\sigma }$, as well as the coefficient for the bid amount $\delta = - \frac{1}{\sigma }$.

WTPs for participants with certain characteristics were then calculated using the average of the explanatory variables:

4 $$\begin{array}{*{20}c} {E\left( {WTP{|}\tilde{x}, \beta } \right) = \tilde{x}{\prime} \left( { - \frac{\alpha }{\delta }} \right)} \\ \end{array}$$

Detailed model specifications and diagnostic tests are provided in Supplementary Table S1. Model selection employed a stepwise approach, beginning with a comprehensive model and refining covariate selection to optimize fit. T-tests compared differences in WTP between common and rare disease scenarios.

## Results

### Demographics of participants

A total of 99 participants were included in the study, yielding a response rate of 82.5%. All invitees responded to our invitation; those who did not participate declined the invitation due to time conflicts. Participants’ demographic and professional characteristics are summarized in Table 1. Most participants (n = 87, 87.88%) originated from eastern China, with notable representation from Beijing (n = 23, 23.23%), Shanghai (n = 27, 27.27%), and Jiangsu Province (n = 13, 13.13%). Additionally, over half (57.57%) had more than ten years of work experience, and approximately half specialized in health insurance (49.49%).

**Table 1:** Background information of participants

Demographic items	n (%)
Affiliation cities/provinces	
Beijing	23 (23.23)
Tianjin	4 (4.04)
Shandong	9 (9.09)
Shanghai	27 (27.27)
Jiangsu	13 (13.13)
Zhejiang	4 (4.04)
Guangdong	4 (4.04)
Fujian	1 (1.01)
Others	14 (14.14)
Affiliation region	
East	87 (87.88)
Middle	4 (4.04)
West	8 (8.08)
Working experience	
< 5 years	19 (19.19)
5–10 years	23 (23.23)
11–20 years	33 (33.33)
> 20 years	24 (24.24)
Expertise (may overlapping)	
Health insurance	49 (49.49)
Pharmacy	22 (22.22)
Pharmacoeconomics	78 (78.79)
Others	5 (5.05)

### Frequencies of WTP intervals

**Fig. 2:**
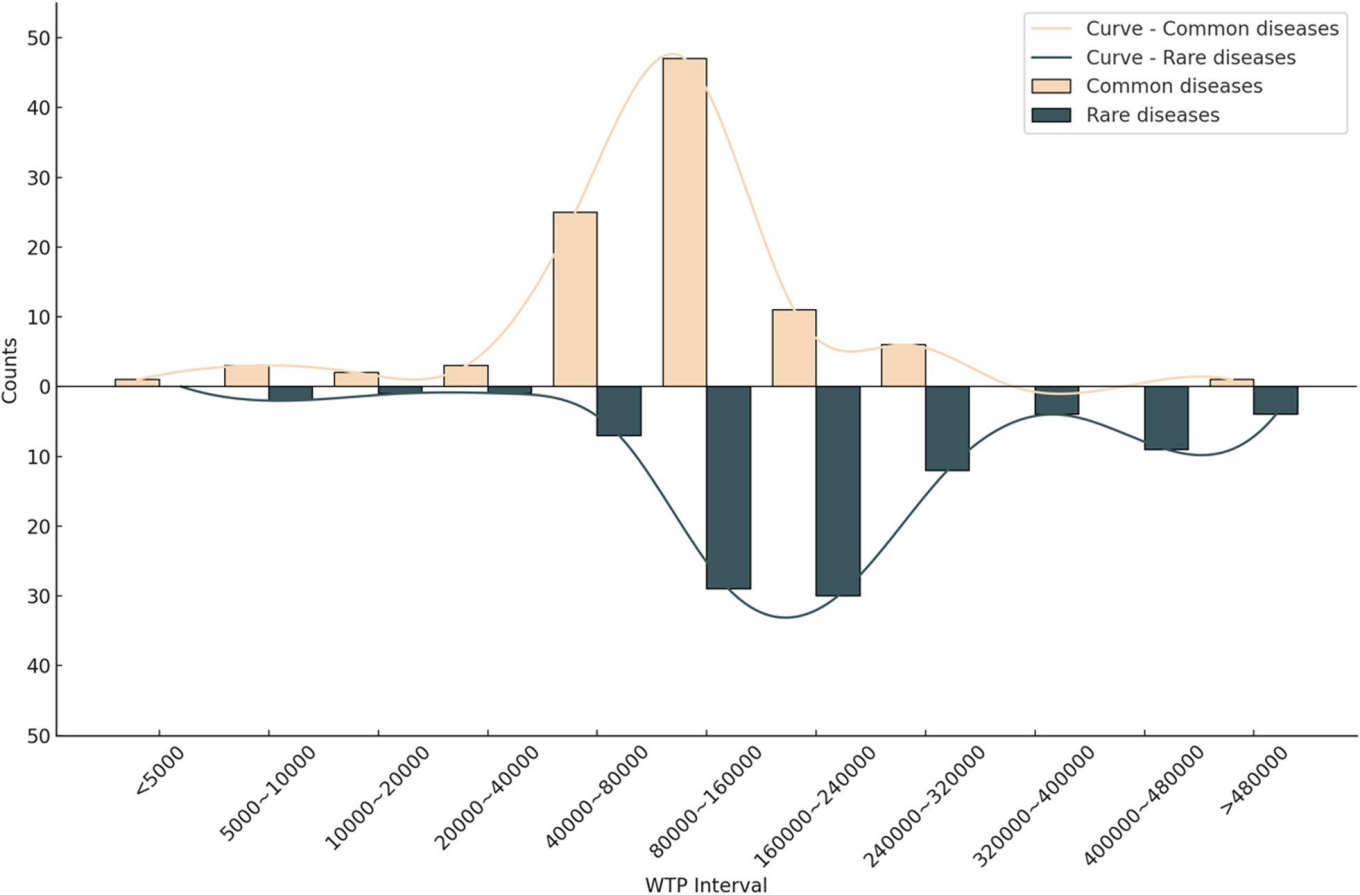
Frequencies of WTP intervals of participants

Participants’ WTP for drug reimbursement in common and rare disease scenarios is presented as frequency distributions of WTP intervals in Fig. 2. Both distributions exhibit an approximately normal (bell-shaped) pattern, but with distinct central tendencies. For common diseases, most people chose amounts in the 40,000–80,000 range. For rare diseases, most people chose higher amounts, mainly in the 80,000–160,000 range. In both cases, fewer people picked very low or very high amounts, and most responses were grouped around the middle.

In the common disease scenario, the majority of participants reported WTP intervals between CNY 40,000–80,000 and CNY 80,000–160,000, with the latter category comprising nearly half of respondents (47.47%). No participants indicated a WTP in the highest interval (CNY 320,000–400,000). For rare diseases, participants primarily reported WTP intervals of CNY 80,000–160,000 (29.29%) and CNY 160,000–240,000 (30.3%).

### WTP per QALY for common diseases

Probit regression results for the common disease scenario are detailed in Table 2. In Model 1 (without covariates), the bid amount showed a significant negative relationship with participants’ willingness-to-pay (*p* < 0.01). The estimated WTP per QALY in this model was CNY 126,449.4 (95% CI 116,603.4–136,295.4, *p* < 0.01), approximately 1.58 times the GDP per capita.

**Table 2:** Probit model and WTP in the common disease scenario

Exploratory variables	Model 1 (without covariates) ^a^		Model 2 (reduced model) ^b^		Model 3 (full model) ^c^	
	Coefficient	95% CI	Coefficient	95% CI	Coefficient	95% CI
Bid	− 0.000013***	(− 0.000015, − 0.000012)	− 0.000014***	(− 0.000015, − 0.000012)	− 0.000014***	(− 0.000015, − 0.000012)
Female	–	–	0.278**	(0.026, 0.529)	0.294**	(0.039, 0.550)
Working experience (senior)	–	–	–	–	0.009	(− 0.275, 0.293)
Expertise in health insurance	–	–	0.240*	(− 0.010, 0.490)	0.288**	(0.015, 0.561)
Expertise Pharmacy	–	–	–	–	0.208	(− 0.150, 0.566)
Expertise in Pharmacoeconomics	–	–	0.386**	(0.085, 0.686)	0.465***	(0.134, 0.795)
South	–	–	–		0.014	(− 0.270, 0.298)
Western	–	–	–		− 0.070	(− 0.477, 0.338)
constant term	1.656***	(1.473, 1.839)	1.148***	(0.831, 1.465)	1.062***	(0.397, 1.728)
WTP	126,449.4***	(116,603.4, 136,295.4)	84,543.21***	(61,501.33, 107,585.1)	78,030.92***	(29,209.58, 126,852.3)

^a^ Model 1 includes response as the explained variable and Bid as the main exploratory variable; no other covariates are included in the model. ^b^ Model 2 is the reduced model, with the lowest AIC and three covariates. ^c^ Model 3 is the full model

^*^p < 0.1; **p < 0.05; ***p < 0.01

The reduced model (Model 2), identified via stepwise regression, included female sex, expertise in health insurance, and expertise in pharmacoeconomics as significant covariates. Specifically, female participants (β = 0.278, 95% CI 0.026–0.529, *p* < 0.05) and participants with pharmacoeconomic expertise (β = 0.386, 95% CI 0.085–0.686, *p* < 0.05) were significantly associated with higher WTP. Model 2 estimated a lower WTP per QALY at CNY 84,543.21 (95% CI 61,501.33–107,585.1, *p* < 0.01), equivalent to 1.06 times GDP per capita.

The full model (Model 3), incorporating all covariates, confirmed significant positive associations for female participants (β = 0.294, 95% CI 0.039–0.550, *p* < 0.05), expertise in health insurance (β = 0.288, 95% CI 0.015–0.561, *p* < 0.05), and expertise in pharmacoeconomics (β = 0.465, 95% CI 0.134–0.795, *p* < 0.01). The estimated WTP per QALY was CNY 78,030.92 (95% CI 29,210.58–126,852.3, *p* < 0.01), corresponding to 0.98 times GDP per capita. Sensitivity analyses and robustness checks are provided in Supplementary Table S2.

### WTP per QALY for rare diseases

Probit regression results for the rare disease scenario are shown in Table 3. In Model 1 (without covariates), the estimated WTP per QALY was CNY 219,691.1 (95% CI 206,480.5–232,901.7, *p* < 0.01), approximately 2.75 times GDP per capita.

**Table 3:** Probit model and WTP in the rare disease scenario

Exploratory variables	Model 1 (without covariates) ^a^		Model 2 (reduced model) ^b^		Model 3 (full model) ^c^	
	Coefficient	95% CI	Coefficient	95% CI	Coefficient	95% CI
Bid	8.45E−06***	(7.64e−06, 9.25e−06)	8.63E−06***	(7.80e−06, 9.46e−06)	8.66E−06***	(7.82e−06, 9.50e−06)
Female	–	–	− 0.199*	(− 0.423, 0.026)	− 0.202*	(− 0.427, 0.024)
Working experience (senior)	–	–	− 0.178	(− 0.418, 0.062)	− 0.201	(− 0.454, 0.051)
Expertise in health insurance	–	–	− 0.189*	(− 0.420, 0.042)	− 0.219*	(− 0.460, 0.022)
Expertise in Pharmacy	–	–	0.271	(− 0.021, 0.563)	0.252	(− 0.062, 0.566)
Expertise in pharmacoeconomics	–	–	–	–	− 0.065	(− 0.358, 0.227)
South	–	–	–	–	− 0.103	(− 0.355, 0.149)
Western	–	–	–	–	0.091	(− 0.272, 0.453)
constant term	− 1.856***	(− 2.044, − 1.668)	− 1.650***	(− 1.927, − 1.373)	− 1.588***	(− 2.195, − 0.980)
WTP	219,691.1***	(206,480.5, 232,901.7)	191,155.8***	(162,576, 219,735.6)	183,391.5***	(114,306.4, 252,476.6)

^a^Model 1 includes response as the explained variable and Bid as the main exploratory variable; no other covariates are included in the model. ^b^Model 2 is the reduced model, with the lowest AIC and four covariates. ^c^Model 3 is the full model

^*^p < 0.1; **p < 0.05; ***p < 0.01

The reduced model (Model 2) included four covariates: female sex, extensive working experience, expertise in health insurance, and expertise in pharmacy. Female participants (β = −0.199, 95% CI − 0.423–0.026, *p* < 0.1) and those with health insurance expertise (β = −0.189, 95% CI − 0.420–0.042, *p* < 0.1) had marginally significant negative associations with WTP. This model estimated a WTP per QALY of CNY 191,155.8 (95% CI 162,576–219,735.6, *p* < 0.01), equivalent to 2.39 times GDP per capita.

In the full model (Model 3), consistent associations were observed, with marginally significant negative relationships for female participants (β = − 0.202, 95% CI − 0.427–0.024, *p* < 0.1) and health insurance expertise (β = −0.219, 95% CI − 0.460–0.022, *p* < 0.1). The estimated WTP per QALY was CNY 183,391.5 (95% CI 114,306–252,471, *p* < 0.01), or approximately 2.29 times GDP per capita.

### Difference in WTP per QALY

Table 4 summarizes the differences in WTP per QALY between the common and rare disease scenarios using models without covariates and the full models. Generally, WTP estimates were significantly higher for rare diseases compared to common diseases.

**Table 4:** Comparison of WTP per QALY between common disease and rare disease scenarios

Model type	Covariates	Disease types	WTP	SE*	95% CI	Differences	*P* value
Probit	No covariate	Common	126,449.4	5023.58	(116,603.4, 136,295.4)	93,241.7	< 0.001
		Rare	219,691.1	6740.22	(206,480.5, 232,901.7)		
	Full model	Common	78,030.92	24,909.3	(29,209.58, 126,852.3)	105,360.58	< 0.001
		Rare	183,391.5	35,248.15	(114,306.4, 252,476.6)		

SE, Standard error

In models without covariates, the WTP difference was CNY 93,241.7 (*p* < 0.001): CNY 126,449.4 (SE: 5023.58, 95% CI 116,603.4–136,295.4) for common diseases versus CNY 219,691.1 (SE: 6740.22, 95% CI 206,480.5–232,901.7) for rare diseases, representing about 1.17 times GDP per capita.

Using full models, the WTP difference increased to CNY 105,360.58 (*p* < 0.001): CNY 78,030.92 (SE: 24,909.3, 95% CI 29,209.58–126,852.3) for common diseases versus CNY 183,391.5 (SE: 35,248.15, 95% CI 114,306.4–252,476.6) for rare diseases, equating to approximately 1.32 times GDP per capita. All differences were statistically significant (*p* < 0.001).

## Discussion

This study is the first to estimate and compare WTP per QALY gained for treatments in end-of-life scenarios involving rare and common diseases, based on the perspectives of health insurance decision makers in China. Using the contingent valuation method, we found that respondents were willing to pay between 0.98- and 1.58-times GDP per capita per QALY for common diseases, and between 2.29- and 2.75-times GDP per capita for rare diseases. The substantial difference in values suggests an explicit recognition of rarity as a relevant consideration in healthcare resource allocation, particularly in life-threatening conditions.

The higher WTP for rare diseases likely reflects broader awareness of the specific challenges they present. These include high treatment costs, limited therapeutic options, and the social disadvantage often experienced by patients [15, 16]. Our findings are consistent with an international policy trend that rarity is treated as an equity-relevant factor in health technology assessment and health care resource allocation decisions. For example, the United Kingdom’s National Institute for Health and Care Excellence allows much higher thresholds for highly specialised technologies used to treat rare diseases [11]. Similarly, Australia’s Life Saving Drugs Program supports access to high-cost treatments for rare, life-threatening conditions even when they fall outside standard reimbursement criteria [17]. Our finding on WTP for rare diseases also aligns with previous empirical evidence derived from trade-off and discrete choice studies [18–20]. Respondents in those studies also prioritised rare disease treatments despite their higher costs. However, the relatively high WTP we observed for rare diseases raises concerns about fiscal sustainability. Future research should examine how cognitive bias, framing effects, and other contextual factors influence valuation in resource allocation decisions.

Our estimates for common diseases are broadly in line with previous studies conducted in upper middle income and high-income countries [21, 22]. For instance, thresholds have been reported at 0.93 times GDP per capita in Argentina, 0.95 in Brazil, and 1.34 in Iran. In the United Kingdom, the standard threshold increases from £20,000 to £30,000 per QALY for most treatments [11], equivalent to roughly 0.5–0.8 times UK GDP per capita, and can rise to £50,000 per QALY (roughly 1.3 GDP per capita) for end-of-life treatments [23].

In the Chinese context, some studies have reported higher estimates than ours for WTP per QALY for common diseases. For example, Xu and colleagues reported 1.76 to 2.06 times GDP per capita [24], and Peng and colleagues estimated 1.86 times GDP per capita for non-small cell lung cancer [25]. Differences may reflect methodological choices. Earlier studies often used basic analytical approaches, such as ordinary least squares or interval regression, whereas we applied probit models to improve precision. Prior studies have frequently relied on samples drawn from the general public [13, 24, 25]. However, given China’s population of 1.4 billion, it is often infeasible to obtain a truly representative sample, which may result in biased estimates of societal preferences. In contrast, our study surveyed nearly the entire population of national-level policymakers responsible for drug reimbursement decisions, providing more policy-relevant and internally valid insights. Moreover, many earlier studies adopted a demand side perspective [5, 13], while our analysis is grounded in the supply side, which reflects opportunity costs under fiscal constraints [26]. From a theoretical perspective, demand side thresholds capture societal preferences and perceived value, whereas supply side thresholds aim to represent the health benefits that would be foregone if resources were allocated elsewhere [27, 28]. Supply side estimates are typically lower, as they are anchored in budget realities, but they are especially useful for informing decisions in publicly funded health insurance systems. [6, 29]

Our findings have several policy implications. First, they support the case for using differentiated cost-effectiveness thresholds, possibly 1.2 to 1.3 times higher for rare diseases than for common conditions. These thresholds could be integrated into ongoing reforms to the National Reimbursement Drug List in China. Second, evidence on higher WTP for rare diseases could inform improvements to China’s Rare Disease Catalogue and National Registry. Specifically, these WTP data can inform the refinement of inclusion criteria for the Rare Disease Catalogue, guide the prioritisation of diseases for coverage based on societal preferences, and support the development of evidence-based budget allocations that better reflect public values. Third, differentiated thresholds may guide pricing negotiations with manufacturers and justify higher reimbursement limits for rare disease treatments. To manage potential fiscal impact, complementary mechanisms such as performance-based payment models, managed entry agreements, or disease-specific funding arrangements should be considered.

This study has several limitations. Although the sample size is modest, it includes most of the national health insurance decision makers in China, which enhances the policy relevance and generalizability of the findings. Second, we applied a single-bounded contingent valuation format and held disease severity constant, focusing specifically on end-of-life scenario. While this approach is intuitive and suitable for policymaking contexts, it limits our ability to precisely capture how multiple equity-related factors interact and influence WTP from supply side. Future studies could employ richer valuation methods, such as double-bounded or discrete choice designs [30–32] and incorporate a broader range of equity attributes (such as disease severity, the novelty of treatments for rare diseases, the potential for catastrophic health expenditures, and the onset age of symptom manifestation) to yield more comprehensive insights into decision-makers'preferences [1, 33]. Finally, our approach implicitly assumes that participants incorporate productivity losses and future medical costs into their valuations; however, these economic impacts were not explicitly assessed. Ignoring explicit consideration of productivity and future healthcare expenditures may lead to incomplete estimates of societal value in health technology assessment [34, 35]. Future research should explicitly incorporate productivity losses and future medical costs caused by rare or common diseases into the design of decision-making scenarios. By presenting policymakers with scenarios that more accurately reflect the full range of economic consequences, studies can generate more realistic and policy-relevant evidence to inform resource allocation decisions.

## Conclusions

This study estimated Chinese health insurance policymakers’ WTP per QALY, finding values ranging from 0.98 to 1.58 times GDP per capita for common diseases and from 2.29 to 2.75 times GDP per capita for rare diseases. Our findings demonstrate that disease rarity significantly influence policymakers’ valuations explicitly within an end-of-life context. This supports the implementation of differentiated cost-effectiveness thresholds specifically tailored to end-of-life treatment scenarios. The results provide an essential reference point for developing informed cost-effectiveness thresholds in China and underscore the necessity of integrating equity considerations—particularly fairness toward patients with rare conditions—into healthcare decision-making. Although derived from hypothetical scenarios, our estimates offer valuable insights for establishing transparent and socially responsive reimbursement policies. Future research should further investigate the underlying factors driving these valuations, explore additional disease attributes that may affect WTP, such as disease severity, the novelty of available treatments, the potential for catastrophic health expenditures, and the typical age of disease onset. Ultimately, this study contributes important evidence supporting a more equitable, evidence-based approach to health technology assessment in China.

## Supplementary Information

Additional file 1.

## Data Availability

The data of the study will be made available on request to the corresponding author on: wanghaiyin@shdrc.org.

## Abbreviations

QALY: Quality-adjusted life year
WTP: Willingness to pay
NHSA: National healthcare security administration
NICE: National institute for health and care excellence

## References

[1] Gu Y, Lancsar E, Ghijben P, Butler JRG, Donaldson C. Attributes and weights in health care priority setting: a systematic review of what counts and to what extent. Soc Sci Med. 2015;146:41–52. 26498059 10.1016/j.socscimed.2015.10.005

[2] Ghijben P, Gu Y, Lancsar E, Zavarsek S. Revealed and stated preferences of decision makers for priority setting in health technology assessment: a systematic review. Pharmacoeconomics. 2018;36(3):323–40. 29124632 10.1007/s40273-017-0586-1

[3] Chen Q, Hoyle M, Jeet V, Gu Y, Sinha K, Parkinson B. Unravelling the association between uncertainties in model-based economic analysis and funding recommendations of medicines in Australia. Pharmacoeconomics. 2025;43(3):283–96. 39546247 10.1007/s40273-024-01446-z PMC11825629

[4] Jiang S, Chen Z, Wu J, Zang X, Jiang Y. Addressing methodological and ethical issues in practicing health economic evaluation in China. J Glob Health. 2020. 10.7189/jogh.10.020322. 33110524 10.7189/jogh.10.020322 PMC7561214

[5] Cai D, Shi S, Jiang S, Si L, Wu J, Jiang Y. Estimation of the cost-effective threshold of a quality-adjusted life year in China based on the value of statistical life. Eur J Health Econ. 2021. 10.1007/s10198-021-01384-z. 34655364 10.1007/s10198-021-01384-z PMC9135816

[6] Ochalek J, Wang H, Gu Y, Lomas J, Cutler H, Jin C. Informing a cost-effectiveness threshold for health technology assessment in China: a marginal productivity approach. Pharmacoeconomics. 2020;38(12):1319–31. 32856280 10.1007/s40273-020-00954-y

[7] McDougall JA, Furnback WE, Wang BCM, Mahlich J. Understanding the global measurement of willingness to pay in health. J Mark Access Heal Policy. 2020;8(1):1717030. 10.1080/20016689.2020.1717030 PMC7048225 32158523

[8] Garber AM, Phelps CE. Economic foundations of cost-effectiveness analysis. J Health Econ. 1997;16(1):1–31. 10167341 10.1016/s0167-6296(96)00506-1

[9] Woods B, Revill P, Sculpher M, Claxton K. Country-level cost-effectiveness thresholds: initial estimates and the need for further research. Value Health. 2016;19(8):929–35. 27987642 10.1016/j.jval.2016.02.017 PMC5193154

[10] Lancsar E, Gu Y, Gyrd-Hansen D, et al. The relative value of different QALY types. J Health Econ. 2020. 10.1016/j.jhealeco.2020.102303. 32061405 10.1016/j.jhealeco.2020.102303

[11] Health NIf, Excellence C. NICE health technology evaluations: the manual. 2022.

[12] Health NIf, Guidance CEC. Eculizumab for treating atypical haemolytic uraemic syndrome. 2015.

[13] Ye Z, Abduhilil R, Huang J, Sun L. Willingness to pay for one additional quality adjusted life year: a population based survey from China. Appl Health Econ Health Policy. 2022;20(6):893–904. 35934772 10.1007/s40258-022-00750-z PMC9358064

[14] Shiroiwa T, Sung YK, Fukuda T, Lang HC, Bae SC, Tsutani K. International survey on willingness-to-pay (WTP) for one additional QALY gained: What is the threshold of cost effectiveness? Health Econ. 2010;19(4):422–37. 19382128 10.1002/hec.1481

[15] Hanchard MS. Debates over orphan drug pricing: a meta-narrative literature review. Orphanet J Rare Dis. 2025;20(1):107. 40055799 10.1186/s13023-025-03634-2 PMC11887186

[16] Jiang S, Wang H, Gu Y. Genome sequencing for newborn screening—an effective approach for tackling rare diseases. JAMA Netw Open. 2023;6(9):e2331141-e. 37656463 10.1001/jamanetworkopen.2023.31141

[17] Taylor C, Jan S, Thompson K. Funding therapies for rare diseases: an ethical dilemma with a potential solution. Aust Health Rev. 2017;42(1):117–9. 10.1071/AH16194 28202130

[18] Bourke SM, Plumpton CO, Hughes DA. Societal preferences for funding orphan drugs in the United Kingdom: an application of person trade-off and discrete choice experiment methods. Value Health. 2018;21(5):538–46. 29753350 10.1016/j.jval.2017.12.026

[19] Magalhaes M. Can severity outweigh smaller numbers? A deliberative perspective from Canada. Value Health. 2018;21(5):532–7. 29753349 10.1016/j.jval.2018.03.010

[20] Schlander M, Telser H, Fischer B, Tv R, Schaefer R, (2018) Drivers of social value exceed length and quality of life: evidence from Switzerland. Value Health. 2018;21:115

[21] Pichon-Riviere A, Drummond M, Palacios A, Garcia-Marti S, Augustovski F. Determining the efficiency path to universal health coverage: cost-effectiveness thresholds for 174 countries based on growth in life expectancy and health expenditures. Lancet Glob Health. 2023;11(6):e833–42. 37202020 10.1016/S2214-109X(23)00162-6

[22] Nimdet K, Chaiyakunapruk N, Vichansavakul K, Ngorsuraches S. A systematic review of studies eliciting willingness-to-pay per quality-adjusted life year: does it justify CE threshold? PLoS ONE. 2015;10(4): e0122760. 25855971 10.1371/journal.pone.0122760 PMC4391853

[23] Health N, Excellence C. Appraising life-extending, end of life treatments. London: National Institute for Health and Clinical Excellence; 2009.

[24] Xu L, Chen M, Angell B, et al. Establishing cost-effectiveness threshold in China: a community survey of willingness to pay for a healthylife year. BMJ Glob Health. 2024;9(1): e013070. 38195152 10.1136/bmjgh-2023-013070 PMC10806867

[25] Peng Q, Yin Y, Liang M, et al. Estimating the cost-effectiveness threshold of advanced non-small cell lung cancer in China using mean opportunity cost and contingent valuation method. Cost Eff Resour Alloc. 2023;21(1):80. 37915053 10.1186/s12962-023-00487-z PMC10621116

[26] Claxton K, Martin S, Soares M, et al. Methods for the estimation of the NICE cost effectiveness threshold. Health Technol Assess. 2015;19(14):1–504. 25692211 10.3310/hta19140 PMC4781395

[27] Vallejo-Torres L, García-Lorenzo B, Castilla I, et al. On the estimation of the cost-effectiveness threshold: why, what, how? Value Health. 2016;19(5):558–66. 27565273 10.1016/j.jval.2016.02.020

[28] Thokala P, Ochalek J, Leech AA, Tong T. Cost-effectiveness thresholds: the past, the present and the future. Pharmacoeconomics. 2018;36(5):509–22. 29427072 10.1007/s40273-017-0606-1

[29] Culyer AJ. Cost-effectiveness thresholds in health care: a bookshelf guide to their meaning and use. Health Econ Policy Law. 2016;11(4):415–32. 26906561 10.1017/S1744133116000049

[30] Jiang S, Gu Y, Yang F, et al. Tertiary hospitals or community clinics? An enquiry into the factors affecting patients’ choice for healthcare facilities in urban China. China Econ Rev. 2020. 10.1016/j.chieco.2020.101538.35058675

[31] Wang L, Liu S, Jiang S, et al. Quantifying benefit-risk trade-offs toward prophylactic treatment among adult patients with hemophilia A in China: discrete choice experiment study. JMIR Public Health Surveill. 2023;9: e45747. 37494098 10.2196/45747 PMC10413247

[32] Wang Y, Zhai P, Zhang Y, Jiang S, Chen G, Li S. Gauging incentive values and expectations (G.I.V.E.) among blood donors for nonmonetary incentives: developing a preference elicitation instrument through qualitative approaches in Shandong, China. Patient Patient Cent Outcomes Res. 2023. 10.1007/s40271-023-00639-6. 10.1007/s40271-023-00639-6 37523066

[33] Jiang S, Li B, Parkinson B, Li S, Gu Y. Aggregate distributional cost-effectiveness analysis: a novel tool for health economic evaluation to inform resource allocation. Global Heal Res Policy. 2025;10(1):17. 10.1186/s41256-025-00415-z PMC12001684 40241192

[34] Jiang S, Wang Y, Si L, et al. Incorporating productivity loss in health economic evaluations: a review of guidelines and practices worldwide for research agenda in China. BMJ Glob Health. 2022;7(8): e009777. 35977755 10.1136/bmjgh-2022-009777 PMC9389102

[35] Jiang S, Wang Y, Zhou J, Jiang Y, Liu GG-E, Wu J. Incorporating future unrelated medical costs in cost-effectiveness analysis in China. BMJ Glob Health. 2021;6(10): e006655. 34702751 10.1136/bmjgh-2021-006655 PMC8549663

