# Supplementary material for: How policymakers value end-of-life treatments for rare and common diseases in China: evidence from a contingent valuation study
Source: Glob Health Res Policy. 2025 Aug 26;10:38. doi: 10.1186/s41256-025-00434-w (PMC12379523; doi:10.1186/s41256-025-00434-w)
Supplement: Supplementary file 1 — Additional file 1. [file 41256_2025_434_MOESM1_ESM.docx]

**Supplementary information**

**Table S1**. Models for the common disease scenario

| **Exploratory variables** | **Model 1 (without covariates)** ^a^ | | **Model 2 (reduced model)** ^b^ | | **Model 3 (full model)** ^c^ | |
| --- | --- | --- | --- | --- | --- | --- |
|  | **Coefficient** | **95% CI** | **Coefficient** | **95% CI** | **Coefficient** | **95% CI** |
| Bid | -0.000026*** | (-0.000029, -0.000022) | -0.000026*** | (-0.000030, -0.000023) | -0.000026*** | (-0.000030, -0.000023) |
| Female | - | - | 0.452* | (-0.026, 0.930) | 0.475* | (-0.011, 0.961) |
| Working experience (senior) | - | - | - | - | -0.052 | (-0.586, 0.483) |
| Expertise in health insurance | - | - | 0.403* | (-0.073, 0.880) | 0.515* | (-0.001, 0.561) |
| Expertise Pharmacy | - | - | - | - | 0.435 | (-0.239, 1.109) |
| Expertise in pharmacoeconomics | - | - | 0.687** | (0.131, 1.243) | 0.836*** | (0.214, 1.458) |
| South | - | - | - |  | 0.062 | (-0.468, 0.592) |
| Western | - | - | - |  | -0.183 | (-0.966, 0.599) |
| constant term | 3.038*** | (2.659, 3.418) | 2.167*** | (1.571, 2.762) | 2.056*** | (0.788, 3.325) |
| WTP | 118875.8*** | (109071.9, 128679.6) | 118963*** | (109304.1, 128621.9) | 118961.4*** | (109331, 128591.7) |

Notes: ^a^ Model 1 includes response as the explained variable and Bid as the main exploratory variable; no other covariates are included in the model. ^b^ Model 2 is the reduced model, with the lowest AIC and three covariates. ^c^ Model 3 is the full model.

**Table S2**. Models for the rare disease scenario

| **Exploratory variables** | **Model 1 (without covariates)** | | **Model 2 (reduced model)** | | **Model 3 (full model)** | |
| --- | --- | --- | --- | --- | --- | --- |
|  | **Coefficient** | **95% CI** | **Coefficient** | **95% CI** | **Coefficient** | **95% CI** |
| Bid | -1.50E-05 | (-0.000017, -0.000013) | -1.54E-05 | (-0.000017, -0.000014) | -1.54E-05 | (-0.000017, -0.000014) |
| Female | - | - | 0.346* | (-0.068, 0.760) | 0.351* | (-0.065, 0.766) |
| Working experience (senior) | - | - | 0.300 | (0.143, 0.743) | 0.323 | (-0.140, 0.786) |
| Expertise in health insurance | - | - | 0.385* | (-0.042, 0.813) | 0.417* | (-0.023, 0.858) |
| Expertise Pharmacy | - | - | -0.496 | (-1.026, 0.033) | -0.475 | (-1.045, 0.095) |
| Expertise in pharmacoeconomics | - | - | - | - | 0.084 | (-0.459, 0.628) |
| South | - | - | - | - | 0.138 | (-0.321, 0.597) |
| Western | - | - | - | - | -0.203 | (-0.871, 0.464) |
| constant term | 3.240*** | (2.867, 3.613) | 2.890*** | (2.372, 3.408) | 2.890*** | (1.762, 4.017) |
| WTP | 214797.4*** | (201108.1, 228486.7) | 214784*** | (201253.6, 228314.4) | 214785.4*** | (201270, 228300.9) |

Notes: ^a^ Model 1 includes response as the explained variable and Bid as the main exploratory variable; no other covariates are included in the model. ^b^ Model 2 is the reduced model, with the lowest AIC and four covariates. ^c^ Model 3 is the full model.
